# Local increase in trapezius muscle oxygenation during and after acupuncture

**DOI:** 10.1186/1476-5918-8-2

**Published:** 2009-03-16

**Authors:** Masaki Ohkubo, Takafumi Hamaoka, Masatugu Niwayama, Norio Murase, Takuya Osada, Ryotaro Kime, Yuko Kurosawa, Ayumi Sakamoto, Toshihito Katsumura

**Affiliations:** 1 Tokyo Therapeutic Institute, 3 Sanei-cho, Shinjuku-ku, Tokyo 160-0008, Japan; 2 National Institute of Fitness and Sports in Kanoya, 1 Shiromizu, Kanoya, Kagoshima 891-2393, Japan; 3 Department of Sports Medicine for Health Promotion Tokyo Medical University, 6-1-1, Shinjuku, Shinjuku-ku Tokyo 160-8402, Japan; 4 Department of Electrical and Electronic Engineering Faculty of Engineering Shizuoka University, 3-5-1 Johoku, Hamamatsu, Shizuoka 432-8561, Japan; 5 Department of Neurology, University of Cincinnati, 3125 Eden Ave, 2327 Vontz Center for Molecular Studies, PO Box 670536, Cincinnati, OH 45267-0536, USA

## Abstract

**Purpose:**

This study aimed to compare the trapezius muscle blood volume and oxygenation in the stimulation region and in a distant region in the same muscle during acupuncture stimulation (AS). We hypothesized that AS provokes a localized increase in muscle blood volume and oxygenation in the stimulation region.

**Methods:**

Two sets of near-infrared spectrometer (NIRS) probes, with 40-mm light-source detector spacing, were placed on the right trapezius muscle, with a 50-mm distance between the probes. Changes in muscle oxygenation (oxy-Hb) and blood volume (t-Hb) in stimulation and distant regions (50 mm away from the stimulation point) were measured using NIRS. Nine healthy acupuncture-experienced subjects were chosen as the experimental (AS) group, and 10 healthy acupuncture-experienced subjects were chosen for the control (no AS) group. Measurements began with a 3-min rest period, followed by "Jakutaku" (AS) for 2 min, and recovery after stimulation.

**Results:**

There was a significant increase in oxy-Hb (60.7 μM at maximum) and t-Hb (48.1 μM at maximum) in the stimulation region compared to the distant region. In the stimulation region, a significant increase in oxy-Hb and t-Hb compared with the pre-stimulation level was first noted at 58.5 s and 13.5 s, respectively, after the onset of stimulation.

**Conclusion:**

In conclusion, oxygenation and blood volume increased, indicating elevated blood flow to the small vessels, not in the distant region used in this study, but in the stimulation region of the trapezius muscle during and after a 2-min AS.

## Background

A considerable number of patients (61.5/1000) who complain of shoulder stiffness (SS) visit oriental therapeutic clinics [1]. Several studies have shown that acupuncture can be a useful modality for treating pain due to muscle spasms [2-4]. It is believed that SS is caused primarily by restriction of blood flow to the working muscles where accumulated metabolites appear to activate sympathetic vasoconstrictors. Acupuncture stimulation (AS) is reported to increase local tissue blood flow in animals [5-7] and humans [3,8,9] and is applicable in therapeutic interventions of SS.

Regarding the propagation of the signal transmitted by AS to the other parts of the muscles, it has been reported that an increase in muscle blood flow was noted only in the muscle where AS was applied [10]. Vasodilatation response was only obtained by stimulation of the dorsal root ipsilateral but not contralateral to the biceps femoris muscle [7]. However, spatial distribution of AS-induced vasodilatation response has never been quantified in a single muscle. In other words, there is no evidence whether the influence of AS would propagate to a region distant from the stimulation point across a single muscle. There are several reports indicating that there exists a latency in C-fibre activation (~5 s) or vasodilatation response (15~20 s) from the onset of AS [11,12]. However, no temporal data has ever been investigated regarding blood flow response at the onset of AS in humans.

Conventional invasive techniques (such as invasive laser Doppler flowmetry) for evaluating muscle blood flow have limitations such as a relatively great burden on subjects and tissue destruction that may influence blood flow itself. Recently, near-infrared spectroscopy (NIRS) has been used for monitoring muscle blood volume and oxygenation in a localized area (~20 cm^3^) with a good temporal resolution (~0.5 s) [13-15]. Therefore, the use of NIRS would present a challenge regarding whether we could detect a temporal response at AS onset and differentiate a regional response between restricted areas of interest modulated by AS.

The purpose of this study was to compare the trapezius muscle blood volume and oxygenation in the stimulation region and in a distant region in the same muscle during acupuncture stimulation (AS) and to estimate a latency at the onset of AS. We hypothesized that AS provokes a localized increase in muscle blood volume and oxygenation in the stimulation region.

## Methods

### Subjects

Nine healthy acupuncture-experienced subjects (2 men and 7 women; average age, 36 years; height and BMI (mean ± SD) 164.4 ± 7.2 cm and 21.0 ± 0.8, respectively) who volunteered from a group of qualified acupuncture therapists were recruited for the experiment as the AS group. Ten healthy acupuncture-experienced subjects (3 men and 7 women; average age, 29 years; height and BMI (mean ± SD) 161.9 ± 7.2 cm and 21.0 ± 3.3, respectively) who volunteered from a group of qualified acupuncture therapists were recruited as the control group (no AS). All participants provided their written consent on a form approved by the institutional ethical committee after receiving complete written and verbal details of the experimental protocol and any potential risks involved. Subcutaneous adipose tissue thickness (ATT) was measured between the light source and detector of the NIRS probe, both in the stimulation and distant regions (5 cm away from the stimulation point) by using an ultrasound device (RT2600; GE Yokogawa Medical Systems). The average ATT was 4.9 ± 1.2 mm (range: 3.0 to 6.0 mm) in the stimulation region and 6.0 ± 2.3 mm (range: 3.0 to 9.0 mm) in the distant region.

### Experimental protocol

**Figure 1:**
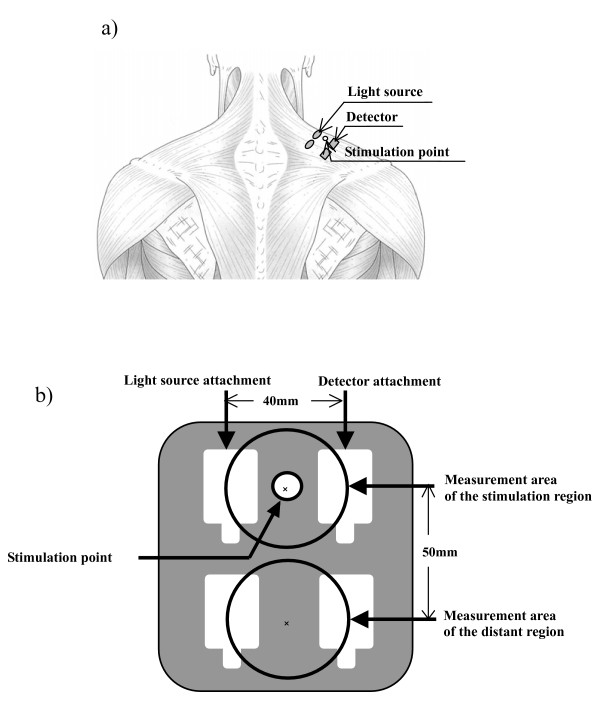
**Measurement site and stimulation point (a) and measurement area and stimulation point (b)**.

The subjects were instructed to fast and refrain from smoking for at least 3 h prior to the experiment. They were asked to maintain natural breathing (10–15 times per min) and stay relaxed and awake throughout the experiment. Prior to starting the experiment, the subjects sat on a chair, with the right upper limb at rest in a vertical position and the left upper limb naturally extended and placed level with the heart. Two sets of NIRS probes were placed on the belly of the right trapezius muscle of the shoulder, with a 50-mm separation between probes (Fig. 1a &1b). We affixed the probe in the measurement area with surgical tape. First, the attachment edge of the probe was affixed. In addition, the probe's light source and detector were more strongly affixed to the measurement area using surgical tape extending from the right frontal area of the chest, over the shoulder and extending into the shoulder blade area. The measurement range was in the hemisphere of 1/2 of the distance between the light source and detector (20 mm) [16]. It is necessary to separate the measurement areas by 40 mm or more to prevent the near-infrared rays interfering when two NIRS are measured simultaneously. Therefore, we assumed a distance of 50 mm in consideration of this issue (Figure 1b). The distance between the paired light input and the detector was set at 40 mm, and the stimulation point was at the centre of the path between the light source and detector. We left a hole of a diameter of 8 mm in the probe between the light source and detector as shown in figure 1b. We covered all windows in the laboratory so that no extraneous light could enter. A fluorescent light was used in an area farthest away from the measurement point. Therefore, we were able to avoid the influence of other near infrared rays on the measurement value. We confirmed beforehand that the measurement of NIRS was not influenced by the creation of the hole. In this experiment, we determined the stimulation point anatomically in the center of the line, which tied up the acromion edge to the seventh cervical spine spinous process. This position is the same as GB21 (Jianjing) unified by WHO/WPRO [17]. The other probe located on the same muscle, 50 mm away from the stimulation region, was provided as the reference point (distant region). The subjects were instructed to report the perceived sensation at the end of AS to evaluate a subjective perception of the stimulation.

### Experimental protocol for the AS

After 3 min of monitored resting, acupuncture was carefully conducted to prevent eliciting any pain or de-qi sensation in the subjects when moving the needle (length, 50 mm and diameter, 0.24 mm) up and down (2–3 mm vertical motions with no rotations) at 1 Hz and a needle insertion depth of 15–20 mm for 2 min. The reason for the choice of this technique without any de-qi sensation was to minimize the influence of the central nervous system. The measurement was continued for 10 min after the start of the experiment. The de-qi sensation is induced by manually twirling the needle after insertion into muscle tissue, and is characterised by a sore, distending, heavy, or numb feeling. There was no AS for the control group throughout the experiment.

### Measurement of muscle tissue oxygenation

Muscle oxygenation and blood volume were measured by a near-infrared spectroscope (Model HEO-200, OMRON Ltd. Inc., Japan). The equipment has a flexible probe consisting of 2 LEDs that emit light at 760 nm or 840 nm. The light can penetrate soft tissue up to approximately 2.0 cm when the detector is at a distance of 4 cm from the radiation source. Relative changes in oxygenated haemoglobin (oxy-Hb), deoxygenated Hb (deoxy-Hb), and total Hb (t-Hb) were calculated by the equation reported in a previous study [18]. Corrected concentrations (mM) considering the ATT for oxy-Hb, deoxy-Hb, and t-Hb were obtained by dividing the concentrations with normalized measurement sensitivity S as shown in the following equation [19]:

S = exp{-(h/A_1_)^2^}-A_2_G(α,β)

where S is the normalized measurement sensitivity; h is the ATT; G (α,β) is the gamma distribution; and the constants A_1_, A_2_, α, and β at a light source-detector separation of 40 mm are 10.91, 1.59, 7.81, and 1.48, respectively.

The values for oxy-Hb, deoxy-Hb, and t-Hb were each averaged to 10-s intervals at rest (0 to 3 min) and after the end of stimulation (5 to 10 min) and averaged to 3-s intervals during stimulation (3 to 5 min).

To examine the kinetics of oxy-Hb and t-Hb at the onset of stimulation, the time constant (Tc) for the increase in oxy-Hb and t-Hb was calculated by fitting the data into the following mono-exponential equation:

y = a(1-e^-kt^)

In this equation, y represents the relative value of oxy-Hb or t-Hb during stimulation; a, the total amount of change in oxy-Hb or t-Hb from the value at pre-stimulation to the value at stimulation; k, the rate constant (1/k = Tc); and t, time.

### Statistical analysis

The values were reported as the mean ± S.D. The statistical analysis was conducted using SPSS 11.5J for Windows (SPSS Inc.). After verifying that each parameter (oxy-Hb, deoxy-Hb, and t-Hb) was not normally distributed (Kolmogorov Smirnov p < 0.05) a non parametric Wilcoxon signed-rank test (p < 0.05) was conducted to confirm the significant difference in oxy-Hb, deoxy-Hb, and t-Hb between stimulation and distant regions. A Dunnet t-test (p < 0.05) was conducted to examine the significant increase in oxy-Hb and t-Hb during stimulation as compared to the pre-stimulation values.

## Results

**Figure 2:**
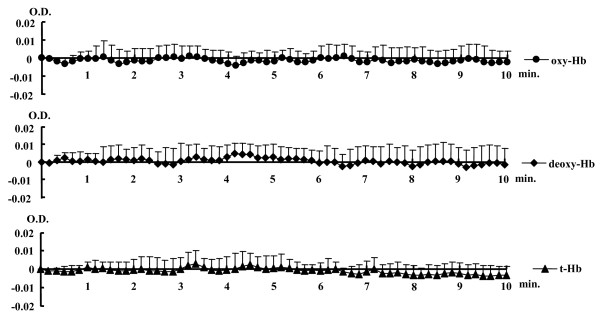
**Changes in oxygenated haemoglobin (oxy-Hb), deoxygenated Hb (deoxy-Hb), and total Hb (t-Hb) in the control group throughout the experiment (no acupuncture stimulation)**. The values for oxy-Hb, deoxy-Hb, and t-Hb in the stimulation region were each averaged to 10-s intervals. There was no change in any parameters for the control group. OD is optical density. The number of the subjects is 10. Values are represented as means ± S.D.

**Figure 3:**
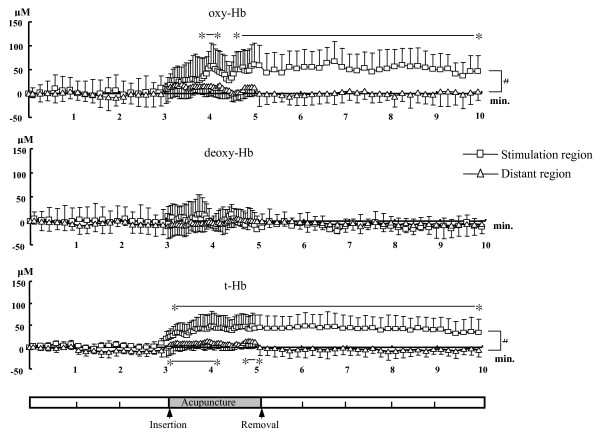
**Changes in oxy-Hb, deoxy-Hb, and t-Hb at pre-stimulation rest and during acupuncture stimulation (AS) and recovery from AS in the stimulation and distant regions in the trapezius muscle**. A significant difference in oxy-Hb and t-Hb is noted between stimulation and distant regions as indicated by (#). Significant increases in oxy-Hb and t-Hb compared with pre-stimulation levels are indicated by (*). The number of the subjects is 9. Values are represented as means ± S.D.

Fig. 2 shows changes in oxy-Hb, deoxy-Hb, and t-Hb in the control group throughout the experiment. There was no change in any parameter for the control group. Fig. 3 shows changes in oxy-Hb, deoxy-Hb, and t-Hb at pre-stimulation rest, during AS, and recovery from AS in the stimulation and distant regions for the AS group. There was a significant difference in the case of both oxy-Hb (60.7 μM at maximum) and t-Hb (48.1 μM at maximum) between stimulation and distant regions. In the stimulation region, a significant increase in oxy-Hb compared with the pre-stimulation level was first noted at 58.5 s after the onset of stimulation and continued thereafter until the end of the observation period, except from 73.5 to 91.5 s. Regarding t-Hb, in the stimulation region, a significant increase compared to the pre-stimulation level was first noted at 13.5 s after the onset of stimulation and continued thereafter until the end of the observation period; in the distant region, it was first noted at 10.5 s and continued thereafter until 145 s after the onset of stimulation, except from 76.5 to 100.5 s. The Tc for the increase in oxy-Hb (46.5 ± 20.0 s) was significantly greater than that for t-Hb (29.2 ± 13.9 s).

## Discussion

We found an increase in oxygenation and blood volume, an indication of elevated blood flow to the small vessels, in the stimulation region of the local trapezius muscle during and after a 2-min AS. The oxygenation and blood volume response was localized to the region where the AS was applied, and there was no response in the same muscle ~3 cm away from the stimulation point.

The increase in oxy-Hb and t-Hb during and after stimulation found in this study indicates enhanced blood flow (oxygen supply) to the small vessels, including arterioles, capillaries, and venules [20]. The enhanced blood flow response induced by AS may be attributable to C-fibre mediated axon reflex [21] resulting from noxious mechanical stimulation. We found a faster response for t-Hb (a significant increase at 14 s after the onset of stimulation and a Tc of 29 s) than for oxy-Hb (a significant increase at 59 s after the onset of stimulation and a Tc of 47 s). A slower response for oxy-Hb suggests that refilling of oxygenated blood into the small vessels, especially to the capillaries and venules, required a certain length of time after vasodilatation of the arterioles and venules where AS-induced vasodilators might directly have an influence. The delayed vasodilatation response (14-s latency for the increase in t-Hb after stimulation) might be due to the nature of the response time for nociceptors and C-fibres. It has been reported that vasodilatation induced by the stimulation of peripheral terminals of nociceptors develops with a latency of 15–20 s and outlasts the time of stimulation [12]. It has also been suggested that C-fibre activation evokes an ~5-s delay from the onset of AS at a very low (1 Hz) frequency of stimulation [11]. The long-lasting effects, even after the termination of stimulation where the needle remains stationary, on hyperoxygenation indicate that vasoactive substances such as calcitonin gene-related peptide (CGRP) from the sensory nerve terminals are involved in the mechanisms [9]. The present study protocol differed from the previous study [9] in that the needle was removed after stimulation. However, we observed elevated muscle oxygenation levels during recovery after stimulation in cases where there was no direct needle insertion, which is similar to the previous study [3]. It is interesting to note that the elevated muscle blood flow was observed in both studies in spite of different acupuncture techniques: with [3] and without (this study) de-qi sensation.

The centre of the probe that was located at a site distant from the stimulation point was 50 mm away from the centre of the probe that was stationed at the site of needle insertion. Since the detector of the NIRS probe used in this study was expected to sense signals 2 cm away from the centre of the light source and the detector, a smaller increase in blood volume in the distant region indicates that there was little vasodilatation response approximately 3 cm away from the needle insertion site. Therefore, it is likely that the nociceptor signal or mechanochemical stimulation induced by the current AS did not conduct to a site at a distance of 3 cm from the site of needle insertion. A plausible explanation of the slight increase in blood volume observed in the distant probe would be that the detector of the distant region was able to detect a slightly larger area than that primarily expected for the distant probe. Although the distribution of C polymodal nociceptors has been examined in the skin of human limbs but not in the muscle [11], the results of this study suggest that the effect of the AS might be, at most, localized ~3 cm away from the AS point or an area ~57 cm^3^. Since it is not clear whether the vasodilation propagated to the other parts of muscle, e, g. lateral or proximal to the stimulation point, multichannel NIRS should be used in future studies [22]. However, an interesting finding in the study by Sandberg et al [3] was a transient significant increase even in contralateral trapezius muscle (which was not stimulated) during AS. This finding might be explained by other mechanisms, possibly central nervous system or mental factors. One might argue that the increases in oxy-Hb and t-Hb found in this study could be due to vasodilatation of the skin. However, since the path of the photons follows the so-called banana-shaped characteristics, the contribution of the skin to the signal should be far less than that of the muscle when an appropriate source-detector spacing of 4 or 5 cm is used [14,16].

Continuous wave NIRS (NIRcws) could not provide absolute values because of unknown physical properties such as the optical path length in tissue [13,20]. Usually, arterial occlusion to the limbs makes it possible to produce zero physiological oxygenation and to compare NIRcws values among varied individuals and different regions [23]. However, since the arterial occlusion method could not be applied to the shoulder muscle, oxygenation could not be quantified in the muscle of SS origination using NIRcws. To compare muscle oxygenation using NIRcws in the trapezius, isometric maximum voluntary contraction (MVC) was used in an attempt to induce maximal deoxygenation in the muscle [24,25]. However, studies on this topic do not entirely address whether the MVC method can create maximal deoxygenation during the contraction and maximal post-contraction hyperemic response consistently in all subjects. There are several studies [13,19,20,26] indicating the effects of ATT on the amplitude of NIRS indicators when the data is expressed in optical density (OD). The larger the ATT, the smaller the NIRS signals [20]. In this study, we observed varied ATT values ranging from 3.0 to 9.0 mm. If the ATT was not adjusted, the NIRS signal drastically differed according to the previous study [19]. The signal sensitivity progressively decreased with increasing ATT, for example, 0.8 for a 3.0-mm ATT and 0.3 for a 9.0-mm ATT when assuming that the sensitivity for zero ATT is 1.0. In other words, we could not compare the signals among regions with different ATTs without adjusting the signal sensitivity primarily arising from the ATT variation. According to a quantitative time-resolved spectroscopy (TRS) data, the hyperemic increase in oxy-Hb and t-Hb after a 12-min arterial occlusion was approximately 20 μM (from 115 μM at rest to 135 μM at peak hyperemia) and approximately 30 μM (from 70 μM at rest to 100 μM at peak hyperemia), respectively [27]. We reported the increase in oxy-Hb (60.7 μM) and t-Hb (48.1 μM), which data might be larger than those obtained by TRS setup where measured t-Hb and oxy-Hb concentration would be diluted due to the adipose tissue layer.

Regarding the effects of blood flow, eliciting the de-qi sensation is superior to merely inserting the needle into the muscle [9]. However, insertion into the muscle without de-qi sensation resulted in a greater increase in blood flow than that into the skin [9]. Painful sensation is not evoked by microstimulation of C-fibre afferents at frequencies less than 2 Hz and even low-discharge frequency stimuli might induce some physiological effects in subjects without any de-qi sensation [10]. Taken together, the stimulation, regarded as low- to moderate-intensity used in this study, is effective for creating a vasodilatation response, presumably with the presence of C-fibre stimuli without any de-qi sensation and pain.

Central circulation is one of the determinant factors for peripheral circulation. In our previous study, we did not find any changes in the parameters related to cardiac output (CO) except for HR (a decrease of 3–8 rpm during AS, but a return to the baseline levels after AS) [28]. Since BP and SNA, indicators of peripheral resistance, and the cardiac sympathetic/parasympathetic activity did not change during AS, it is speculated that the CO remained relatively constant during and after AS. Further, muscle oxygenation levels remained elevated after AS, whereas the HR returned to the pre-AS resting levels following the decrease during AS. Therefore, there is no influence of the central circulation on the response of local muscle oxygenation changes during and after AS.

## Conclusion

In this study, vasodilative substances induced by AS, mediated by the axon reflex via polymodal receptors in the skeletal muscle and the skin, played a role in the local muscle oxygenation response to the stimulation. The oxygenation and blood volume response was localized to the region where AS was applied.

NIRS is able to provide an objective indication for examining the degree of vasodilatation (hyperoxygenation) response. Monitoring NIRS indications would be useful, in particular, for treating patients with shoulder stiffness or muscle spasms to determine the optimal intensity and frequency of AS.

## Competing interests

The authors declare that they have no competing interests.

## Authors' contributions

MO performed selection of the experiment subjects as well as the measurement and evaluation of NIRS data. TH served as the general administrator for the experiment and performed the cast immobilization process. NM and TO performed selection and medical checks of the experiment subjects. YK, KE and MN performed measurement and evaluation of NIRS data. TK and AS examined medical checks of the experiment subjects. All authors read and approved the final manuscript.
